# Negative Smad Expression and Regulation in the Developing Chick Limb

**DOI:** 10.1371/journal.pone.0005173

**Published:** 2009-04-08

**Authors:** Neil Vargesson, Ed Laufer

**Affiliations:** 1 Department of Genetics and Development, Columbia University, New York, New York, United States of America; 2 Department of Pathology and Cell Biology, Columbia University, New York, New York, United States of America; The Rockefeller University, United States of America

## Abstract

The inhibitory or negative Smads, Smad6 and Smad7, block TGFβ superfamily signals of both the BMP and TGFβ classes by antagonizing the intracellular signal transduction machinery. We report the cloning of one *Smad6* and two *Smad7* (*Smad7a* and *Smad7b*) chick homologs and their expression and regulation in the developing limb. *Smad6* and *Smad7a* are expressed in dynamic patterns reflecting the domains of BMP gene expression in the limb. Activation and inhibition of the BMP signaling pathway in limb mesenchyme indicates that negative Smad gene expression is regulated, at least in part, by BMP family signals.

## Introduction

Bone morphogenetic proteins (BMP) are members of the transforming growth factor-β (TGFβ) ligand superfamily. BMPs have diverse essential roles during limb development which include establishment of the apical ridge and zone of polarizing activity, as well as in the regulation of cell death, chrondrogenesis, myogenesis, digit identity and fracture repair [1]–[12]. TGFb/Activin signaling is also implicated in digit tip-specification [5]. These roles necessitate precise control both of ligand expression and the responsiveness of target cells, neither of which is well understood. The negative Smad genes (*Smad6* and *Smad7*), antagonize and block TGFβ superfamily signaling [13]–[16]. Thus, negative Smad activity could influence the spatial and temporal extent, as well as the magnitude, of BMP or TGFβ/Activin signaling during limb development.

In previous studies limited descriptions of *Smad6* expression in the developing chick limb have been reported [5], [17]–[19]. *Smad6* mRNA expression was also found to be upregulated in interdigital regions following Bmp5 protein application [19]. However more detailed analyses that include developmental timecourses of *Smad7* expression or the dependence of negative Smad expression on BMP signaling have not been described. Thus while some information is available about *Smad6* in the developing limb, how the negative Smad gene expression patterns and their expression levels are regulated, and how they might contribute to the dynamic control of BMP or TGFβ/Activin signaling during limb development remains to be determined.

## Results and Discussion

### Cloning of chicken negative Smad genes

**Figure 1:**
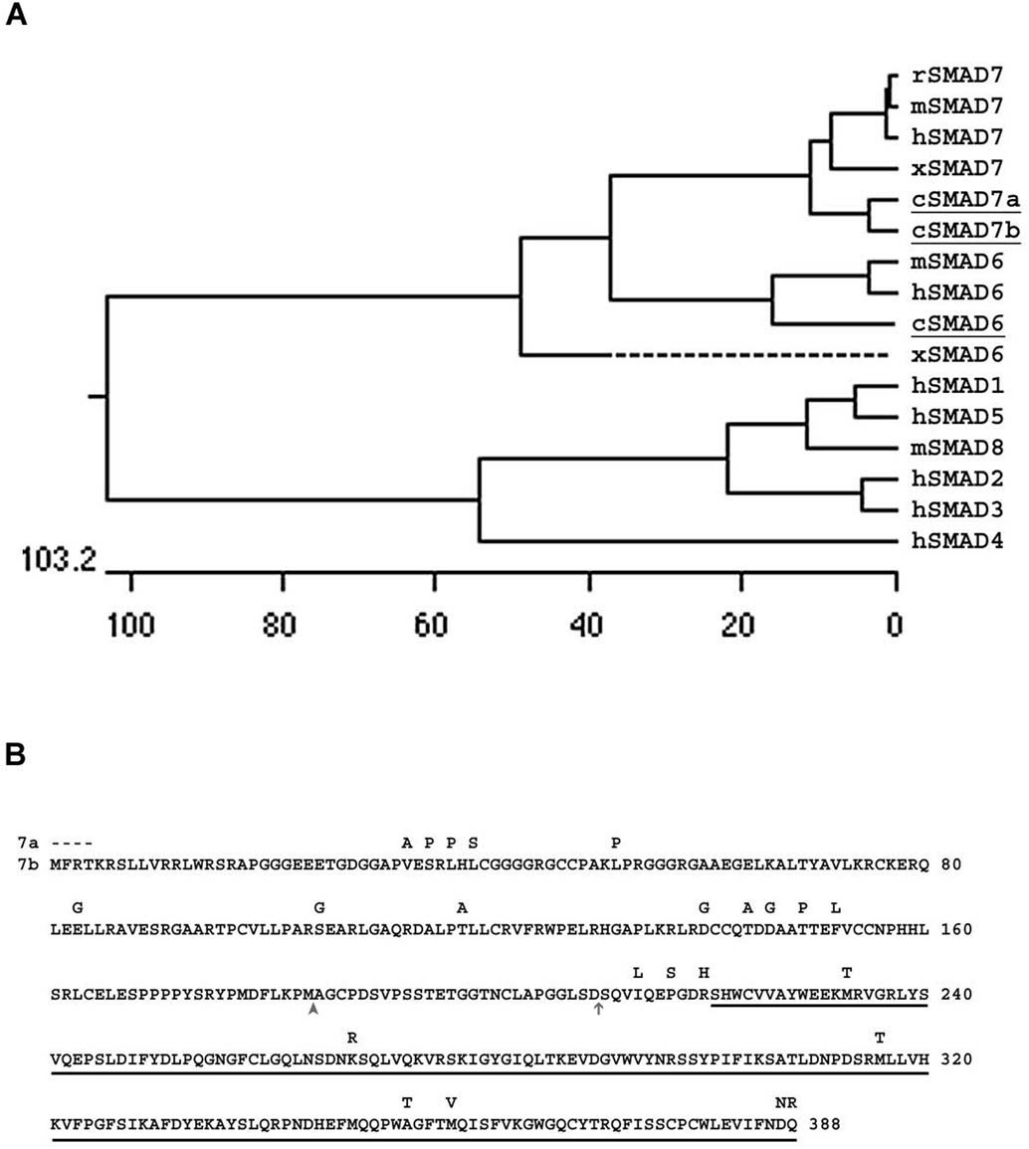
Chicken negative Smad genes. A. Phylogenetic relationship of chicken negative Smad proteins to other Smads. Conceptual translations of the chicken *Smad6*, *Smad7a* and *Smad7b* gene open reading frames were compared to those of other vertebrate negative Smad, positive Smad and common Smad genes. The chick gene names are underlined. x: *Xenopus*, m: mouse, h: human, r: rat, c: chick. B. Comparison of *cSmad7a* (7a) and *cSmad7b* (7b) translated open reading frames. Blank positions in the *cSmad7a* sequence are identical to *cSmad7b*, dashes are absent and differences are indicated. Vertical arrowhead marks the beginning, and vertical arrow marks the end, of the translated sequence conserved between cSmad7a and genomic contig NW_001482773.1. The vertical arrow marks the beginning of the translated sequence conserved between cSmad7a and the genomic cSmad7 locus on the Z chromosome. Underline indicates the MH2 domain that is conserved amongst all Smad proteins. Note absence of serine residues among last four amino acids.

In order to study negative Smad gene function in chick limb bud development, we cloned homologs of *Smad6* and *Smad7* from HH st12–15 whole chicken embryo and HH st20–24 chicken limb bud cDNA libraries. Multiple cDNAs encoding three distinct open reading frames were identified. Comparison with known Smad protein sequences indicates that one encodes a *Smad6* homolog (Genbank Accession FJ417094), while the other two encode *Smad7* homologs (*cSmad7a* and *cSmad7b*; Genbank Accession FJ417093 and FJ417092 respectively; Figure 1). The *cSmad6* gene sequence is identical to that previously described [18]. The two *Smad7* cDNAs are equally related to other *Smad7* genes, and are themselves 93% identical at the nucleotide level within their open reading frames and 80% identical in their 3′ untranslated regions. The conceptual translations of their open reading frames are 98% identical. Neither *Smad7a* nor *Smad7b* has major deviations from other *Smad7* amino acid sequences, and both terminate in a conserved c-terminal motif lacking phosphorylatable serines.

Most vertebrate species have only one *Smad7* homolog. Therefore we compared the *Smad7a* and *Smad7b* sequences with cDNA and chicken genomic sequences present in public databases to ask whether either or both were described previously. The *Smad7b* cDNA contains a complete open reading frame that encompasses a previously described *cSmad7* partial open reading frame (Genbank Accession AF230192), and maps to a contig that is not associated with a particular chromosome within the sequenced chicken genome (Contig: NW_001472907.1). The *Smad7a* cDNA contains a partial open reading frame that is lacking only the four amino terminal amino acids as determined by comparison with other Smad7 proteins. This sequence maps to the predicted genomic *Smad7* locus on the Z chromosome (XM_427238). Thus there are two chicken *Smad7* loci: the cDNA we call *Smad7a* is equivalent to a genomic ‘*Smad7*’ locus on the Z chromosome. The sequence we call *Smad7b* is equivalent to a previously reported ‘*Smad7*’ cDNA, which is not encoded by the genomic *Smad7* locus.

BLAST analysis of *cSmad7a* sequences with public databases identified two distinct genomic sequences with similarities to *cSmad7a*. Nucleotides 541–616 of *cSmad7a* are identical to sequences in an unassigned 1363 nucleotide genomic contig (Contig: NW_001482773.1), while *cSmad7a* nucleotides 615–1235 and genomic DNA sequences at the ‘*Smad7*’ locus have only one mismatch. These data suggest the cDNA spans at least two genomic fragments. Analysis of the conceptual protein sequences is consistent with this idea: The *cSmad7a* ORF encodes a protein of at least 384 amino acids, while the predicted genomic cSmad7 protein sequence is 222 amino acids long. cSmad7a aa207–384 (the translation of nt615–1235) and genomic cSmad7 aa45–222 are identical, consistent with the nucleotide alignments. Protein BLAST analysis of aa1–44 of genomic cSmad7 does not identify any protein other than genomic cSmad7, while similar analysis of cSmad7a aa1–206 or the unassigned genomic contig (NW_001482773.1) identifies numerous Smad7 homologs. These data imply that the first 45 aa predicted by the conceptual translation of genomic *cSmad7* are incorrect, and that the unassigned contig belongs in the genomic sequence in this region.

As cSmad7a is similar to cSmad7b through their most amino terminal amino acids, and genomic regions of identity to *cSmad7a* extend 5′ only to *cSmad7a* nucleotide 541, there is likely at least one additional sequence missing from the genomic sequence. The *cSmad7a* and *cSmad7b* cDNAs show multiple differences scattered throughout their sequences, and are most closely conserved with sequences at different chromosomal locations. Thus *cSmad7a* and *cSmad7b* are encoded by different genes, and are not splice or allelic variants. To the best of our knowledge, the chicken is the first example of an amniote species with two Smad7 genes.

### Negative Smad gene expression during limb development

#### *cSmad6* Expression

**Figure 2:**
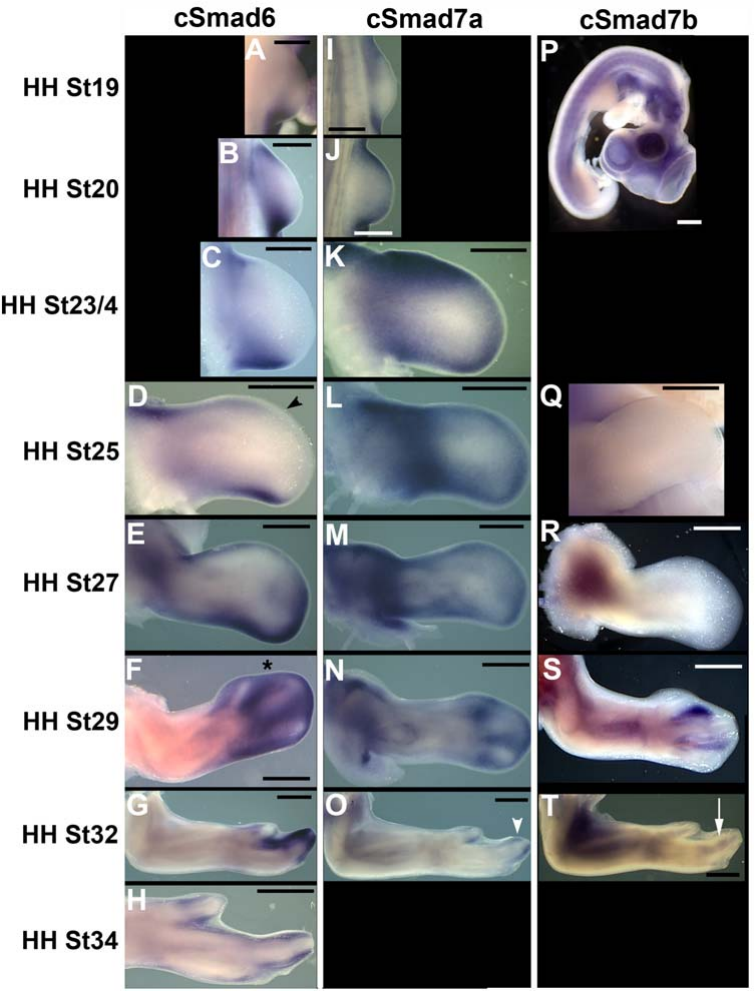
Negative Smad gene expression patterns during chick forelimb development. Expression patterns of *cSmad6* (A–H), *cSmad7a* (I–O) and *cSmad7b* (P–T) in the developing chick forelimb. Developmental stages as indicated. Note *cSmad6* expression domain extends as development proceeds to beneath apical ridge (black arrowhead). Expression of both *cSmad6* and *cSmad7a* is present in distal mesenchyme before being restricted to interdigital regions (black asterisk), then further restricted to areas around the digit tip (white arrowhead). *cSmad7b* is not expressed in the forelimb mesenchyme until HH st29/30 and is then restricted to the cartilage condensations (white arrow). Signal in body and head in (P) is non-specific background. Images are of limb dorsal surface. Anterior to the top; distal to the right. Scale bars indicate 500 µm.

We examined *cSmad6* expression by whole mount in situ hybridization from HH stages 18–34, focusing on the developing limbs. At HH st18 expression is observed in the fore and hindlimb buds in small anterior and posterior mesenchymal domains (Figure 2A–C). As limb outgrowth proceeds expression extends around the distal mesenchymal margin directly abutting the ectoderm and apical ridge (Figure 2D). Weak expression is also detected in the apical ridge through approximately HH st25 (data not shown). As limb outgrowth continues distal mesenchymal expression intensifies beneath the apical ridge, and is maintained along the anterior and posterior margins. From HH st28 *cSmad6* expression becomes restricted to the autopod, primarily in interdigital regions (Figure 2E, F). From HH st32 autopodal expression is peridigitally restricted (Figure 2G, H).

**Figure 3:**
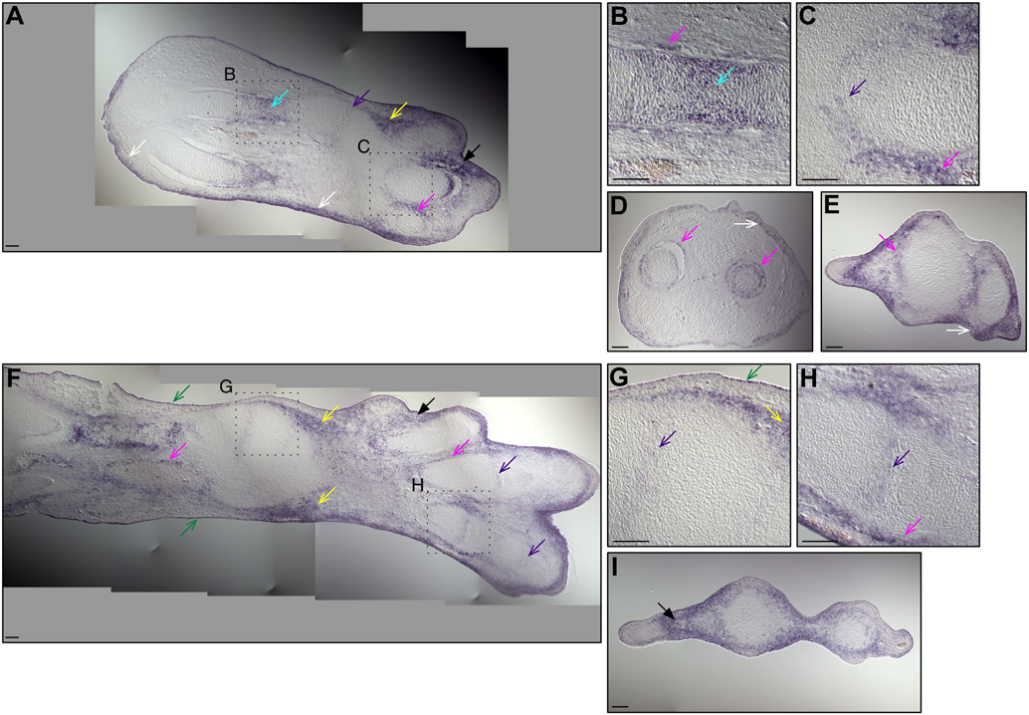
*cSmad6* is expressed in limb mesenchyme, epidermis and cartilage. Expression of *cSmad6* mRNA in longitudinal (A–C, F–H) and transverse (D, F, I) sections of HH st29 forelimbs (A–E) and hindlimbs (F–I). B, C, G, H are higher power images of labeled areas in A and F. Arrows indicate expression of *cSmad6* in interdigital mesenchyme (black arrow); hypertrophic chondrocytes (blue arrow); perichondrium/mesenchyme adjacent to cartilage (magenta arrow); nascent joints (purple arrow); interstitial mesenchyme (yellow arrow); subepidermal mesenchyme (white arrow); epidermis (green arrow). Scale bars indicate 100 µm.

We also examined *cSmad6* expression by section in situ hybridization on HH st29 forelimb (Figure 3A–E) and hindlimb (Figure 3F–I) cryosectioned tissue. We detected expression in interdigital mesenchyme (Figure 3A,F,I), perichrondrium (Figure 3B,D,F), hypertrophic chondrocytes (Figure 3A,B), forming joints (Figure 3A,F–H), interstitial mesenchyme (Figure 3A,F,G), subepidermal mesenchyme (Figure 3A,D,E) and epidermis (Figure 3F,G).

*cSmad6* is expressed in other regions of the developing chick embryo including the heart, feather buds, neural tube, vascular endothelium and facial primordia, including the branchial arches and nasal placodes (data not shown). These data are consistent with, and extend, previously described *cSmad6* expression patterns [18].

#### *cSmad7* Expression

We examined *cSmad7a* and *cSmad7b* expression by whole mount in situ hybridization from HH stages 17–34, again focusing primarily on expression in the developing limb bud. *cSmad7a* is expressed at HH st19 in the lateral mesoderm extending from a small mesenchymal domain in the posterior forelimb to the anterior hindlimb (data not shown; Figure 2I). Expression is also observed in a small mesenchymal domain in the anterior forelimb (Figure 2I,J). As limb outgrowth proceeds, expression of *cSmad7*a, like that of *cSmad6*, extends around the distal margin of the forelimb beneath the apical ridge (Figure 2K,L). From HH st27 transcripts are restricted to interdigital regions of the handplate as well as to posterior marginal mesenchyme (Figure 2M,N). From HH st32 weak expression is observed around the distal tips of the developing digits (Figure 2O). Expression of *cSmad7a* is not apparent at HH st34. *cSmad7a* is also expressed in the developing lungs, heart outflow tract, liver, vascular endothelium, neural tube and gastrointestinal tract of the chick embryo (data not shown). We could not detect significant expression of *cSmad7b* in the developing limbs until HH st29/30 when expression was observed in interdigital mesenchyme and between the radius and ulna (Figure 2P–S). c*Smad7b* expression became restricted to the cartilage condensations at HH st32 (Figure 2T). c*Smad7b* is also expressed in the heart outflow tract from HH st25 (data not shown).

#### Negative Smads are expressed in regions of highest BMP expression

**Figure 4:**
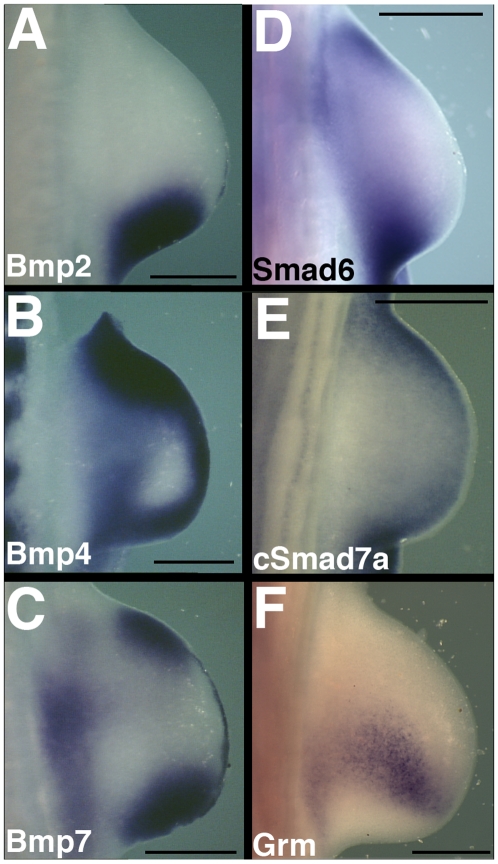
Negative Smad gene expression is restricted to areas of high BMP expression. Expression of BMP and negative Smad genes in HH St20 forelimb buds. *Bmp2* (A), *Bmp4* (B), *Bmp7* (C), *cSmad6* (D), *cSmad7a* (E), *Gremlin* (F). All images show dorsal surface. Anterior to the top, distal to right. Scale bars 500 µm.

Since BMP family signals are potential regulators of negative Smad gene expression, we compared BMP and BMP antagonist gene expression patterns to those of *cSmad6* and *cSmad7a*. We focused on *cSmad7a* rather than *cSmad7b*, as *cSmad7a* is expressed in the limb from HH st 19 through at least HH st32. Taken together the *Bmp2*, *Bmp4* and *Bmp7* expression domains bear a remarkable similarity to those of *cSmad6* and *cSmad7a* (Figure 4A–F; data not shown) [20]. For example, in early limb development the BMP genes, *cSmad6* and *cSmad7a* are expressed in peripheral, but not central, mesenchyme (Figure 4A–E), and later each is expressed interdigitally. In contrast, *Gremlin*, a BMP-dependent antagonist of BMP signaling essential for maintaining signaling pathways regulating limb patterning, is expressed in central mesenchyme (Figure 4F) [21]–[25].

### Regulation of *Smad6* and *Smad7a* expression in the developing limb

**Figure 5:**
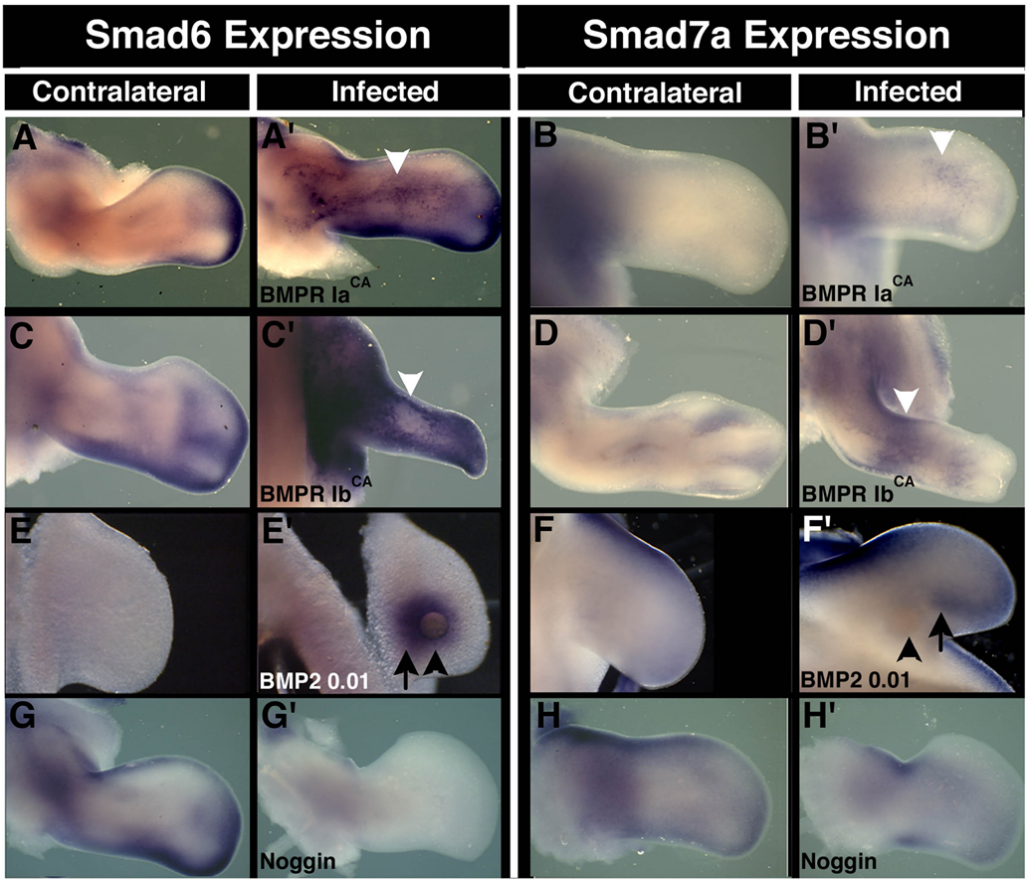
Regulation of negative Smad gene expression by BMP signaling. Expression patterns of *cSmad6* in contralateral (A, C, E, G) or manipulated (A', C', E', G') limbs and of *cSmad7a* in contralateral (B, D, F, H) or manipulated (B', D', F', H') limbs. (A'–D', G'–H') virus infection at HH St17–20, fixed after 48 hr. (E'–F') BMP2 protein soaked bead implanted into proximal mesenchyme at HH St20, fixed at 15 hrs. Experimental manipulation as indicated on panels. Note ectopic expression of *cSmad6* and *cSmad7a* following *BMPR Ia^CA^* and *BMPR Ib^CA^* misexpression (white arrowheads); position of heparin sulphate bead (black arrowhead) and resulting ectopic expression (black arrow). Reduction in limb size in C' and D' is due to cell death induction caused by *BMPR Ia^CA^* misexpression and is not a photographic artifact. All images show dorsal surface. Anterior to top, distal to right. Scale bars 500 µm.

These gene expression patterns led us to test whether negative Smad gene expression might be regulated by BMP signals in the limb. We used retroviral misexpression and recombinant protein application approaches to modulate BMP signaling in vivo. To ectopically activate the BMP signaling pathway in the limb, we infected limb tissue with RCAS retroviruses that express constitutively active forms of the type I BMP receptors, *BMPR Ia* and *BMPR Ib* (*BMPR Ia^CA^* and *BMPR Ib^CA^*) or applied recombinant BMP protein directly to limb tissue [26]. HH st19–20 limb buds were infected with the activated receptor viruses and *cSmad6* and *cSmad7a* expression was assessed up to 72 hours later. Using a virus-specific probe to monitor the extent of infection, we observed extensive but incomplete staining by 24 hours post-infection, and that by 48 hours post-infection had spread throughout the limb mesenchyme (data not shown). In the majority of cases both *cSmad6* and *cSmad7a* were upregulated in their normal expression domains after 24 hr and were also ectopically induced in limb territories such as the central and proximal mesenchyme (Figure 5D; Table 1). Heparin beads soaked in recombinant BMP2 protein were grafted into HH st20 posterior proximal limb mesenchyme and the embryos were harvested 12–15 hours later. Expression of *cSmad6* is induced surrounding the implanted bead at levels higher than those detected in the normal expression domain (Figure 5E; 6 embryos affected of 7 tested; n = 6/7). *cSmad7a* expression is also induced adjacent to the implanted beads, although to a lesser extent than *cSmad6* (Figure 5F; n = 5/7). These data indicate that the negative Smad gene expression can be induced throughout the limb mesenchyme by activating the BMP signaling pathway.

**Table 1:** Summary of virus effects on negative Smad gene expression.

Virus	Undetectable	*cSmad6* expression			*cSmad7a* expression		
		Normal	Reduced	Ectopic	Normal	Reduced	Ectopic
*BMPR Ia^CA^*	-	-	-	15/15	1/7	-	6/7
*BMPR Ib^CA^*	-	4/27	7/27	16/27		3/9	6/9
*Noggin*	9/13	-	4/13	-	2/10	8/10	-
*BMPR Ib^DN^*	-	9/15	6/15	-	4/8	4/8	-

Number of limbs displaying result/total number of limbs examined.

To test whether negative Smad gene expression in the limb is dependent on BMP signals, we infected developing limb tissue with RCAS viruses that express either a dominant negative *BMPR Ib* gene (*BMPR Ib^DN^*) or the BMP signaling antagonist noggin [2], [26]. *BMPR Ib^DN^* virus infection down regulates, but does not completely abolish both *cSmad6* and *cSmad7a* expression (data not shown; Table 1). Viral misexpression of *noggin* reduces expression of *cSmad6* to undetectable levels by 48 hours post-infection (n = 9/13; Figure 5G). In contrast while *cSmad7a* expression is reduced, it is still detectable, even at 72 hours post-infection (n = 8/10; Figure 5H). Thus *Smad6* expression in the chick limb mesenchyme apparently depends completely on BMP signaling, while *Smad7a* expression is partially dependent on similar signals.

## Materials and Methods

### Ethics Statement

Animals were handled in accordance with Columbia University guidelines.

### Cloning and Sequencing

Chicken *Smad6* and *Smad7* homologs were obtained by screening Hamburger and Hamilton (HH) st12–15 chicken embryo [27], [28] and st20–24 chicken limb cDNA libraries [29], [30] with probes derived from mouse and Xenopus *Smad6* and *Smad7* genes [31], [32].

Overlapping clones for each gene were sequenced using standard dye termination chemistry. DNA and protein sequences were compared to the non-redundant GenBank databases and published Smad sequences using both NCBI Blast [33] and DNAStar MegAlign v4.00 software. Genomic comparisons were made to the *Gallus gallus* Genome Build 2.1. Sequences with the following Genbank accession numbers were used to generate the phylogenetic tree in Figure 1: hSMAD1: Q15797, hSMAD2: Q15796, hSMAD3: Q92940, hSMAD4: S71811, hSMAD5: Q99717, hSMAD6: Q43541, mSmad6: AF010133, xSmad6: AF035529, hSMAD7: AAB81354, mSmad7: 2460040, rSmad7: AAC25062: xSmad7: AAC09303, mSmad8: AAF77079. Putative chicken *Smad6* and *Smad7a* and *Smad7b* gene assignments were made based on similarities of the predicted protein sequences with published family members and sequences submitted to Genbank. Accession number for *cSmad6*: FJ417094; *cSmad7a*: FJ417093; *cSmad7b*: FJ417092.

### Embryology and in situ hybridization

Fertile White Leghorn chicken eggs (SPAFAS, Farmington, CT) were incubated at 38°C in a humidified, forced air incubator and embryos collected at appropriate developmental stages. Experimental manipulations were performed on HH st15 to HH st21 right limb buds; the left limb bud served as a control. Embryos were harvested through 72 hr postmanipulation and were fixed overnight in 4% paraformaldehyde. Embryos were processed for non-radioactive whole-mount or section in situ hybridization (20 µm cryosections) and photographed as described [34], [35]. In situ hybridization probes for the chicken genes derived from one clone incorporated into each contig were used. Their names and lengths are: *cSmad6*, Clone 6, 1.7 kb; *cSmad7a*, Clone 10, 2.7 kb; *cSmad7b* Clone 33, 1.0 kb.

### Virology

The RCAS BP(A)*noggin*, RCAS BP(A)*BMPR Ia^CA^*, RCAS BP(A)*BMPR Ib^DN^* and RCAS BP(A)*BMPR Ib^CA^* viruses were described previously and caused phenotypic defects consistent with previously published reports [2], [26]. Concentrated virus stocks were generated using standard procedures, and all stocks had titers of at least 5×10^8^ infectious units/ml [36].

<1?twb=.35w?>For limb infections, virus was injected into HH st17–21 wing buds. Injections were targeted such that either the entire limb or specific subregions were infected with virus. Targeting was monitored by nonradioactive whole mount in situ hybridization using either a pan-retroviral or an insert-specific in situ probe [37].

### Heparin bead preparation and implantation

Heparin acrylic beads were soaked in 0.01 mg/ml recombinant human BMP2 or BMP7 protein for 1–2 hours. A tungsten needle was used to cut a small slit in the posterior proximal mesenchyme of HH st17–20 limbs, and a bead implanted into the slit. Embryos were incubated for approximately 15 hours, fixed in 4% paraformaldehyde and processed for in situ hybridization analysis.
